# Long noncoding RNA SNHG17: a novel molecule in human cancers

**DOI:** 10.1186/s12935-022-02529-7

**Published:** 2022-03-05

**Authors:** Li Ma, Jin Gao, Niu Zhang, Jiawei Wang, Tianwei Xu, Tianyao Lei, Xiaoteng Zou, Chenchen Wei, Zhaoxia Wang

**Affiliations:** 1 Cancer Medical Center, The Second Affiliated Hospital of Nanjing Medical University, Nanjing, 210011 Jiangsu China; 2 Department of General Surgery, The Second Affiliated Hospital of Nanjing Medical University, Nanjing, 210011 Jiangsu China

**Keywords:** SNHG17, lncRNA, Cancer, Gene regulation, Diagnosis, Prognosis

## Abstract

Many studies in recent years have found that dysregulation of long non-coding RNAs (lncRNAs) can contribute to disease. Small nucleolar RNA host gene 17 (SNHG17) is a novel cancer-related lncRNA of the SNHG family which is highly expressed in various tumors and may exert oncogenic functions. Several studies have demonstrated that SNHG17 is closely related to the proliferation, migration, invasion, apoptosis, and chemical drug resistance of tumor cells, and clinical studies have found an association between high SNHG17 expression and poor prognosis. In this review, we summarize relevant studies investigating SNHG17, focusing on its biological function as well as its potential value for clinical applications.

## Introduction

The World Health Organization (WHO) GLOBOCAN 2020 data [1] reported 19.3 million new cancer cases as well as an estimated 10 million cancer deaths worldwide in 2020. These numbers are expected to rise further over the upcoming years, with epidemiologists predicting 34 million new cases worldwide by 2070 [2]. The economic cost related to treatment and nursing costs of cancer patients poses a significant socioeconomic burden and cancer represents a serious issue for human health and social welfare systems [3]. There is an urgent need to improve cancer prevention strategies and develop new early screening and treatment approaches which may help not only increase lifespan but also improve patients’ quality of life and reduce the global socioeconomic burden of cancer.

Long non-coding RNAs (lncRNAs) refer to RNAs of more than 200 bases in length that do not encode proteins, although some new reports [4] suggest that certain ncRNAs may encode micropeptides. In recent years, the small nucleolar RNA host gene (SNHG) family of lncRNAs [5–7] has been demonstrated to exert oncogenic features in various cancers. SNHGs have been found to be overexpressed in several cancer types and may be involved in proliferation, apoptosis, invasion, metastasis, drug resistance and other biological functions of cancer cells. Mechanistically, SNHGs have been reported to mediate their actions as competing endogenous RNAs (ceRNAs). CeRNAs were first described by Salmena et al. [8] in 2011 and correspond to a variety of RNA transcripts with microRNA (miRNA) response elements (MRE). When these transcripts sponge miRNA they can affect the binding of other transcripts to this miRNA, thus regulating the gene expression (9). As shown in our previous study, the LINC01234/miR-204-5p/CBFB axis promotes cells proliferation in gastric cancer (GC) [10].

**Fig. 1:**
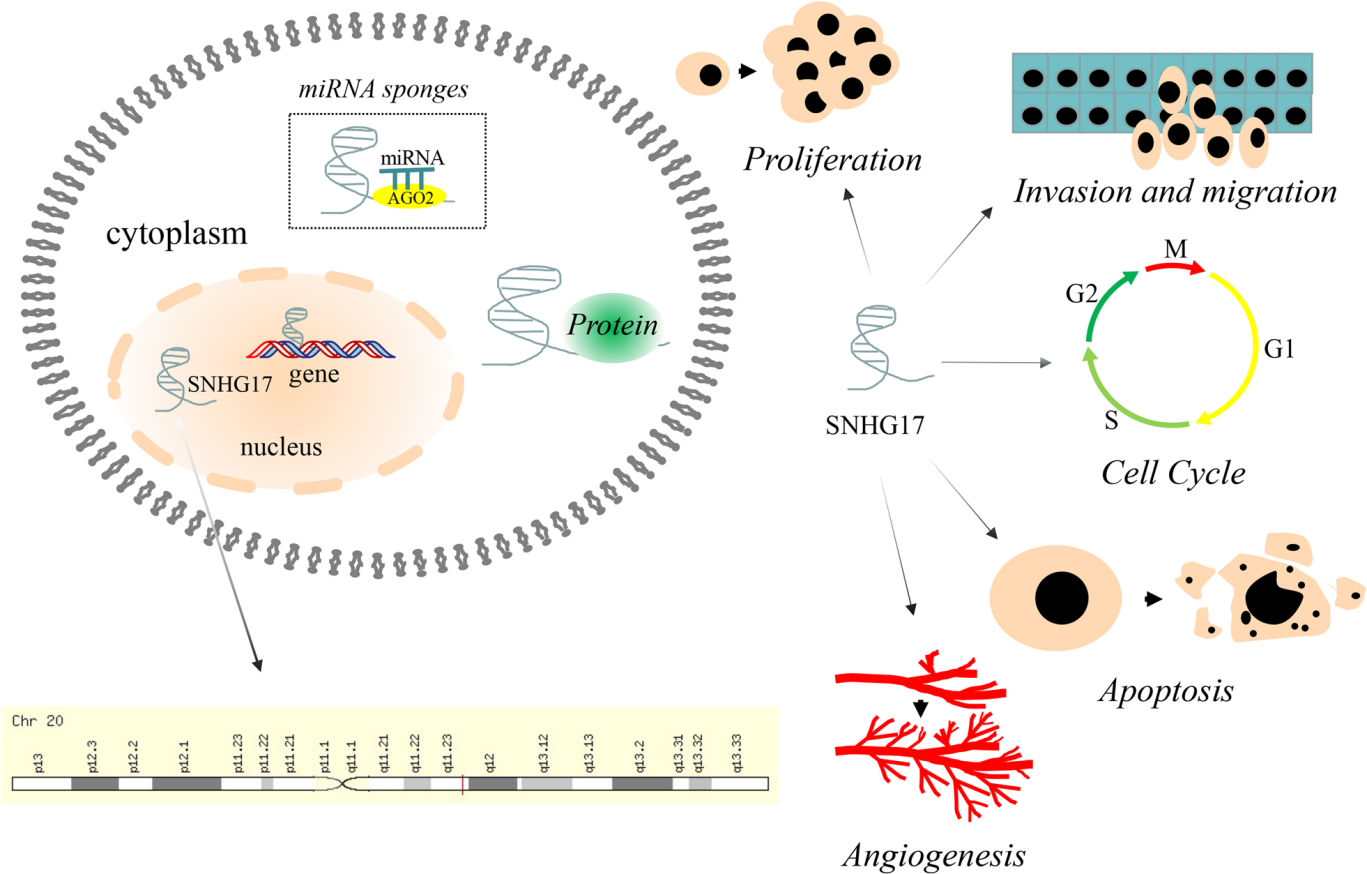
lncRNA SNHG17 location and its functions in tumor cell. SNHG17 locates on human chromosome 20q11.23. SNHG17 performs its function via sponging miRNAs or binding proteins directly. SNHG17 has been reported as an oncogene by regulating cell proliferation, invasion, migration, angiogenesis, cell cycle and apoptosis

Small nucleolar RNA host gene 17 (SNHG17), a 1186 nucleotide lncRNA, is a member of the SNHG family and located on human chromosome 20q11.23. Similar to other members of the SNHG family, it has been reported as an oncogene and can promote proliferation, invasion, migration, and angiogenesis while inhibiting apoptosis in tumors (Fig. 1). SNHG17 has several transcripts and has been shown to be expressed both in the nucleus and cytoplasm of different tumor cells via fluorescence in situ hybridization (FISH) or nucleo-cytoplasmic separation experiment [11, 12]. The first report on SNHG17 was published by Ma et al. [13] and found that SNHG17 overexpression in colorectal cancer promoted cell proliferation via epigenetic silencing of P57. SNHG17 has subsequently also been found to be overexpressed in lung cancer [14], GC [15], hepatocellular carcinoma [16], osteosarcoma [17, 18] and other tumors, and its expression levels have been reported to be associated with a poor prognosis. In addition, SNHG17 also performs unique biological functions in other diseases, such as gestational or type 2 diabetes mellitus [19, 20] and diabetic nephropathy [21]. The above results indicate that SNHG17 may play an essential pathogenic role in a variety of diseases, in particular cancers.

**Fig. 2:**
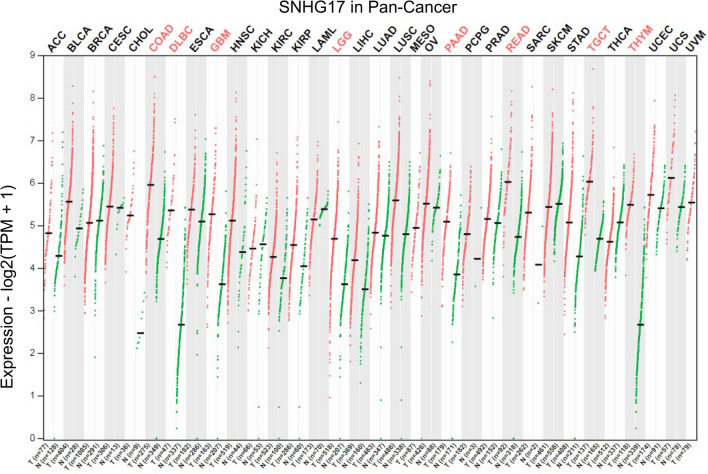
lncRNA SNHG17 expression in various cancers. Data from GEPIA2 database indicates that SNHG17 overexpressed in various tumors, such as colon adenocarcinoma (COAD), esophageal carcinoma, pancreatic adenocarcinoma, rectum adenocarcinoma and so on

While many members of the SHNG family have been systematically reviewed in detail, including SNHG1 [22], SNHG3 [23], SNHG4 [24] and others [25–36], this has not been the case for SNHG17. Data from the Gene Expression Profiling Interactive Analysis 2 (GEPIA2) database indicated that SNHG17 exhibits high pan-cancer expression (Fig. 2). Combined with existing reports on SNHG17, here we summarize the tumor-promoting mechanisms and corresponding clinical significance of SNHG17 in some relative frequent cancer types, such as oral tumor, esophagus cancer, non-small cell lung cancer (NSCLC), breast cancer (BC), GC, colorectal cancer (CRC), hepatocellular carcinoma (HCC), pancreatic cancer, renal cell carcinoma, prostate cancer, ovarian cancer, glioma and melanoma.

## The role of SNHG17 in cancers

### Oral tumors

Oral tumors are highly malignant and have become increasingly prevalent in younger patients in recent years. The main treatment options include surgery, radiotherapy, and chemotherapy, which can further damage the quality of life of patients [37]. The below studies indicate that SNHG17 may play an important role in oral tumors and therefore possesses an important clinical value for the diagnosis and treatment of oral tumors.

Oral squamous cell carcinoma (OSCC) is a highly malignant oral tumor. Without timely and effective treatment, it frequently leads to deformities and death. The 5-year survival rate of OSCC is 55%, with a recurrence rate of 38% [38]. SNHG17 has been found to be significantly overexpressed both in OSCC cell lines [39] and clinical samples of OSCC patients compared to controls, and its expression levels were positively correlated with adverse pathological indicators [40]. Qiao et al. [39] investigated the biological mechanism of SNHG17 in OSCC through various techniques including RNA Binding Protein Immunoprecipitation (RIP), RNA pulldown, chromatin immunoprecipitation (ChIP), and luciferase reporter assay and so on, they reported that SNHG17 acted as a ceRNA of miR-384. SNHG17 sponged miR-384 to upregulate ELF1, a transcriptional activator of CTNNB1, This resulted in activation of the Wnt/β-catenin signaling pathway and could promote the development and occurrence of OSCC. In line with these results, Tong et al. [40] revealed that overexpressed SNHG17 promoted the proliferation, migration, and invasion of OSCC cells while inhibiting their apoptosis, Mechanically, SNHG17 acted as a ceRNA of miR-375 and upregulated PAX6 to exert its carcinogenic role.

Tongue squamous cell carcinoma (TSCC) is the most common subtype of oral cancer. TSCC usually leads to masticatory and speech dysfunction and dysphagia, seriously affecting the quality of life of patients [41]. The incidence and mortality of TSCC in China have been reported to be 4.81% and 2.21%, respectively [42]. Quantitative real-time quantitative polymerase chain reaction (qRT-PCR) analysis of 56 paired TSCC and adjacent normal tissues indicated that SNHG17 was highly expressed in TSCC tissues. Its expression was furthermore positively correlated with advanced tumour-node-metastasis (TNM) stages and shorter overall survival (OS) [41]. Liu et al. [41] revealed that SNHG17 promoted cells proliferation, migration, and invasion of TSCC tumor cells in vitro as well as tumor metastasis in vivo*,* by sponging miR-876 and subsequent upregulation of SP1.

### Esophagus cancer

Esophagus cancer is a common gastrointestinal tumor [1]. Esophageal squamous cell carcinoma (ESCC) accounts for approximately 90% of esophageal cancers in China [43], and its 5-year survival rate lies below 30% [44]. Chen et al. [45] found that SNHG17 was upregulated in 126 carcinoma tissues compared to adjacent non-cancerous tissues, the same result in cells. Their analysis [45] also revealed that SNGH17 promoted epithelial-mesenchymal transition (EMT) progress, proliferation and invasion of ESCC cells by sponging miR-338-3p, thereby activating SOX4, These data suggested that SNHG17 accelerates ESCC progression through miR-338-3P/SOX4 pathways.

### Non-small cell lung cancer

Lung cancer is a major contributor to new cancer cases and deaths, and poses a serious threat to human health [46], around 85% of them is NSCLC [47]. SNHG17 was reported to be upregulated in both NSCLC tissues and cell lines [14, 48, 49] and associated with advanced TNM stage and shorter OS [49]. SNHG17 knockdown has moreover been found to inhibit NSCLC cell proliferation, migration, invasion and EMT progress, while promoting cell apoptosis in vitro*.* Our group [14] previously revealed that high expression of SNHG17 in NSCLC was a result of gene amplification. Gene expression analysis in SNHG17 knockdown cells revealed that SNHG17 inhibits FOXA1 while upregulating XAF1 and BIK, three genes closely related to cell proliferation and apoptosis. Researches from other groups moreover found that high SNHG17 expression promotes lung adenocarcinoma (LUAD) cell proliferation, migration and invasion via sponging miR-485-5p [48] and miR-193a-5p [49]. The authors concluded that SNHG17 promotes LUAD development via the miR-485-5p/WLS [48] and miR-193a-5p/NETO2 [49] pathways. Taken together, these studies suggest an important role for SNHG17 in regulating proliferation, migration and invasion in NSCLC, and it may represent a potential target for diagnosis and treatment to NSCLC.

### Breast cancer

According to the latest statistical data [1], BC has now surpassed lung cancer as the cancer with the highest incidence, and it also represents the main cause of cancer deaths worldwide. Using qRT-PCR, Du et al. [12] assessed that SNHG17 is also upregulated in BC tissues and cells compared with controls, their data also suggested that upregulated SNHG17 predicts advanced TNM stages and lower survival time for patients with BC. In their research, SNHG17 expedites BC cell proliferation, migration, and invasion by targeting miR-485-5p, but further research is required to elucidate the exact mechanisms of action of SNHG17 in BC.

### Gastric cancer

GC is a common malignant tumor with more than 1 million new cases worldwide each year. Unfortunately, most patients are diagnosed with advanced stages at the first clinical visit and the median survival of advanced patients treated with chemotherapy is less than one year [50]. There is an urgent need for new, accurate, and convenient detection techniques to diagnose GC at early stages.

Studies revealed that SNHG17 is significantly upregulated in both GC tissues [15, 51] and cells [15]. Data by Chen et al. [51] suggested that high expression of SNHG17 is not only positively correlated with advanced TNM stages, lymph node metastasis, and distant metastasis, but also negatively correlated with progression-free survival (PFS) and OS in GC patients. Multivariate analyses suggested that SNHG17 might serve as an independent prognostic marker for GC. Zhang et al. [15] found that SNHG17 expression levels has a high diagnostic value for GC, with an area under the receiver operating characteristic (ROC) curve (AUC) of 0.748 (P < 0.001). In their report [15], SNHG17 depletion inhibited cell proliferation, arrested the cell cycle in the G0/G1 phase, promoted apoptosis, and suppressed cell migration and invasion both in vitro* and vivo.* Mechanistically, SNHG17 acted as an oncogene in GC by epigenetically silencing *p*15 and *p*57 via activation of EZH2.

Helicobacter pylori (HP) infection is thought to be the main cause of gastric cancer [50]. Using a lncRNA microarray, Han et al. [11] identified SNHG17 closely related to HP-infected gastric, its expression was gradually increased with increased gastric mucosal lesion degree. Mechanically, SNHG17 knockdown reduced the level of DNA double-strand breaks (DSBs) following HP infection via binding to NONO, a molecule which could upregulate the expression of the DNA damage repair protein Ku80. The authors [11] additionally revealed that SNHG17 could alter the DNA repair system via SNHG17-miR-3909-RING1/Rad51 axis in GC. Taken together, these results indicate that SNHG17 acts as an oncogene in GC and could represent a novel biomarker for its diagnosis, treatment, and prognosis.

### Colorectal cancer

CRC is a malignant tumor with high morbidity and mortality, accounting for approximately 10% of new cases and deaths per year [1]. About 20% of CRC patients present with distant metastasis at the time of diagnosis, most of which are found in the liver and lung. The 5-year survival rate of metastatic CRC patients is only 14%, and surgical treatment or systemic therapy offer little survival benefit to patients [52]. It is therefore particularly urgent to explore the pathogenesis and identify novel targets for early diagnosis and treatment.

Data from Gene Expression Omnibus (GEO) (GSE21510) [13], Encyclopedia of RNA Interactomes (ENCORI) and GEPIA databases [53] suggests that SNHG17 is highly expressed in CRC tissues. Further evidence for an upregulation of SNHG17 in CRC stems from qRT-PCR analysis in CRC tissues from clinical tissue samples [13, 53, 54] or CRC cells [13, 53–55]. SNHG17 expression is also positively correlated with tumor size and TNM stages, while it is negatively correlated with CRC prognosis [13]. High SNHG17 expression may accelerate CRC cell proliferation, invasion, migration and inhibit apoptosis. SNHG17 has been reported to function as miRNA sponges of miR-375 [53], miR-23a-3p [55], miR-361-3p [54]. Liu et al. [53] demonstrated the role of miR-375 in CRC, they illustrated that miR-375, a direct target of SNHG17, could reverse the inhibitory effect of SNHG17 knockdown on EMT progression by targeting CBX3. In Huang’s study [54], they suggested that SNHG17 sponges miR-361-3p to upregulate STC2, which is considered to promote proliferation of CRC cells. While Liu’s report [55] showed that SNHG17 could ptomote the metastasis of CRC via sponging miR-23a-3p, inducing an upregulated expression of CXCL12, a molecule which could regulate tumor angiogenesis. In addition, Ma et al. [13] proved that SNHG17 could epigenetically silence p57 transcription via interaction with EZH2 in CRCs.

Via several experimental approaches, the above studies propose SNHG17 as an oncogene in CRC. At the same time, SNHG17 may have an important clinical value for the prediction of prognosis. Therefore, SNHG17 may not only represent a novel target for CRC treatment but could also be employed for diagnosis and prognostic assessment.

### Hepatocellular carcinoma

Liver cancer is the third leading cause of cancer deaths globally [1]. HCC is the major type of liver cancer, accounting for approximately 90% of liver cancers globally [56] and nearly 50% of liver cancer cases in China [57]. This discrepancy is likely related to a high burden of chronic hepatitis in China [58]. Several studies previously assessed levels of SNHG17 in human HCCs and adjacent normal tissues via qRT-PCR [16, 59, 60] and found a significant upregulation of SNHG17 in cancer tissues. Ma et al. [16] showed that overexpressed SNHG17 promoted the proliferation, migration, invasion and EMT progression of HCC cells. Mechanistic research demonstrated that SNHG17 regulates the miR-3180-3p/RFX1 axis. Meanwhile, Liu et al. [59] found that SNHG17 could combine with LRPRC to decrease the levels of ubiquitylated c-Myc and increase the stability of c-Myc protein, promoting cellular G1/S transition and enhancing cell proliferation.

### Pancreatic cancer

Pancreatic cancer is known for its high malignancy and poor prognosis. Due to its particular anatomical site, early detection remains relatively challenging [61]. High expression of SNHG17 and low expression of miR-942 in pancreatic cancer tissues and cells was discovered by Zhao et al. [62]. Their data revealed that SNHG17 knockdown significantly depressed cell proliferation, viability, migration and invasion, while facilitating apoptosis, this effect could be reversed following overexpression of miR-942. Further research into the target genes of miR-942 will be required to fully elucidate the role of SNHG17 and miR-942 in pancreatic cancer.

### Renal cell carcinoma

Using data from The Cancer Genome Atlas (TCGA) database, Xuan et al. [63] proposed that SNHG17 functions as a regulator of autophagy by regulating ATG4B and CAPN10, although they do not perform any functional experiments. Wu et al. [64] found the higher expression of SNHG17 in renal cell carcinoma tissues and cells compared with controls via qRT-PCR. SNHG17 knockdown significantly inhibited the proliferation, migration, and invasion of tumor cells. The authors reported that SNHG17 may regulate H2AX signaling via miR-328-3p in renal cell carcinoma.

### Prostate cancer

While the incidence of prostate cancer remains high [1], fortunately mortality rates are decreasing year by year [65]. Nonetheless, many patients experience post-treatment sequelae which can seriously affect their quality of life. The exploration and transformation of precise early diagnostic strategies and therapeutic targets are therefore critical.

Reports [66, 67] suggested that SNHG17 is significantly upregulated in both prostate cancer tissues and cells, especially in aggressive and metastatic cells [66]. Moreover, SNHG17 expression is negatively associated with prognosis. In Zhao’s research [66], SNHG17 knockdown inhibited cellular proliferation, invasion, and chemotherapeutic resistance, while facilitating apoptosis. Mechanistic studies indicated that SNHG17 may regulate prostate cancer development via the Wnt/β-catenin pathway. Wu et al. [67] showed that SNHG17 could upregulate its cognate small nucleolar RNA (snoRNA) SNORA71B and thereby promote the proliferation, invasion, migration, and EMT of prostate cancer cells, while inhibiting apoptosis. Mechanistic research suggested that both SNHG17 and SNORA71B transcription was activated by STAT5A. SNHG17 could in turn upregulate the expression of STAT5A by sponging miR-339-3p, thereby affecting SNORA71B expression in a positive feedback loop. In addition, Bai et al. [68] discovered higher expression levels of SNHG17 in tissues from castration resistant prostate cancer (CRPC) than in hormone sensitive prostate cancer (HSPC). Functionally and mechanically, SNHG17 promotes cell proliferation, migration and invasion by competitive binding of miR-144 and upregulation of CD51 in CRPC.

### Ovarian cancer

Ovarian cancer is a common female tumor with an extremely poor prognosis. Unfortunately, a majority of patients relapse after treatments [69]. Studies on effective diagnostic and prognostic markers may help to improve patient survival.

SNHG17 is highly expressed in ovarian cancer tissues and cells [70, 71]. A study investigating data [72] from the TCGA database identified SNHG17 as an autophagy-related gene in ovarian cancer. Moreover, increased SNHG17 expression was associated with shorter OS. In Pan’s study [70], SNHG17 has further been found to suppress apoptosis and promote ovarian cancer cell proliferation in vitro and vivo. Mechanically, transcription factor STAT3 directly binds to the promoter region of SNHG17 and promotes its transcription, and SNHG17 subsequently regulates cell cycle progression and proliferation of ovarian cancer cells via the miR-214-3p/CDK6 axis. Zheng et al. [71] observed that both SNHG17 and FOXA1 are upregulated in ovarian cancer consistently. Functionally, SNHG17 knockdown dampened the proliferative and invasive abilities of cancer cells via partial downregulation of FOXA1. However, the specific mechanism by which SNHG17 regulates FOXA1 remains to be clarified.

### Cervical cancer

Cao et al. [73] reported a high expression of SNHG17 in cervical cancer cells and serum of cervical cancer patients. SNHG17 knockdown significantly depressed proliferation, migration and invasion while facilitating apoptosis of cervical cancer cells. This effect could be reversed by overexpressing miR-375-3p.

### Glioma

Gliomas are the most common primary malignant tumors of the brain. Unfortunately, most patients have an extremely poor prognosis [74], with the exception of circumscribed glioma patients which can be cured by surgical resection. Due to the blood–brain barrier and other factors, chemical drug treatment for gliomas remains difficult and novel therapeutics are required.

Analysis of the GEPIA database [75, 76] revealed a significant upregulation of SNHG17 in glioma compared to control samples. The expression of SNHG17 was moreover found to be negatively associated with OS. Additional studies [75–77] revealed that SNHG17 is highly expressed in both glioma tissues and cell lines, and its expression is associated with malignant features of gliomas. Functional experiments [75, 76] showed that SNHG17 knockdown significantly inhibited the proliferation, migration, and invasion of glioma cells, while promoting apoptosis. Mechanically, Ge et al. [75] proposed that SNHG17 exerts its carcinogenic role via the miR-23b-3p/ZHX1 axis in glioma. Another study by Li et al. [76] suggested that transcription factor YY1 directly binds to the promoter region of SNHG17 and enhances its transcription. SNHG17 subsequently regulates cell proliferation of glioma cells via the miR-506-3p/CTNNB1/Wnt/β-catenin signaling pathway. In addition, Du et al. [77] proposed that SNHG17 could regulate cell proliferation and stemness via the miR-876-5p/ERLIN2 axis in astrocytoma.

### Melanoma

Using qRT-PCR analysis, Gao et al. [78] found that SNHG17 is highly expressed in both melanoma tissues and cell lines. SNHG17 upregulation is positively correlated with lymph node metastasis and decreased OS in melanoma patients, and SNHG17 expression is an independent predictor of melanoma prognosis. Functional studies confirmed that SNHG17 knockdown resulted in significantly reduced proliferation, migration, and invasion of melanoma cells. Mechanistic studies indicated that STAT3-induced upregulation of SNHG17 contributed to melanoma progression by promoting PI3K-AKT signaling.

## Conclusion

**Fig. 3:**
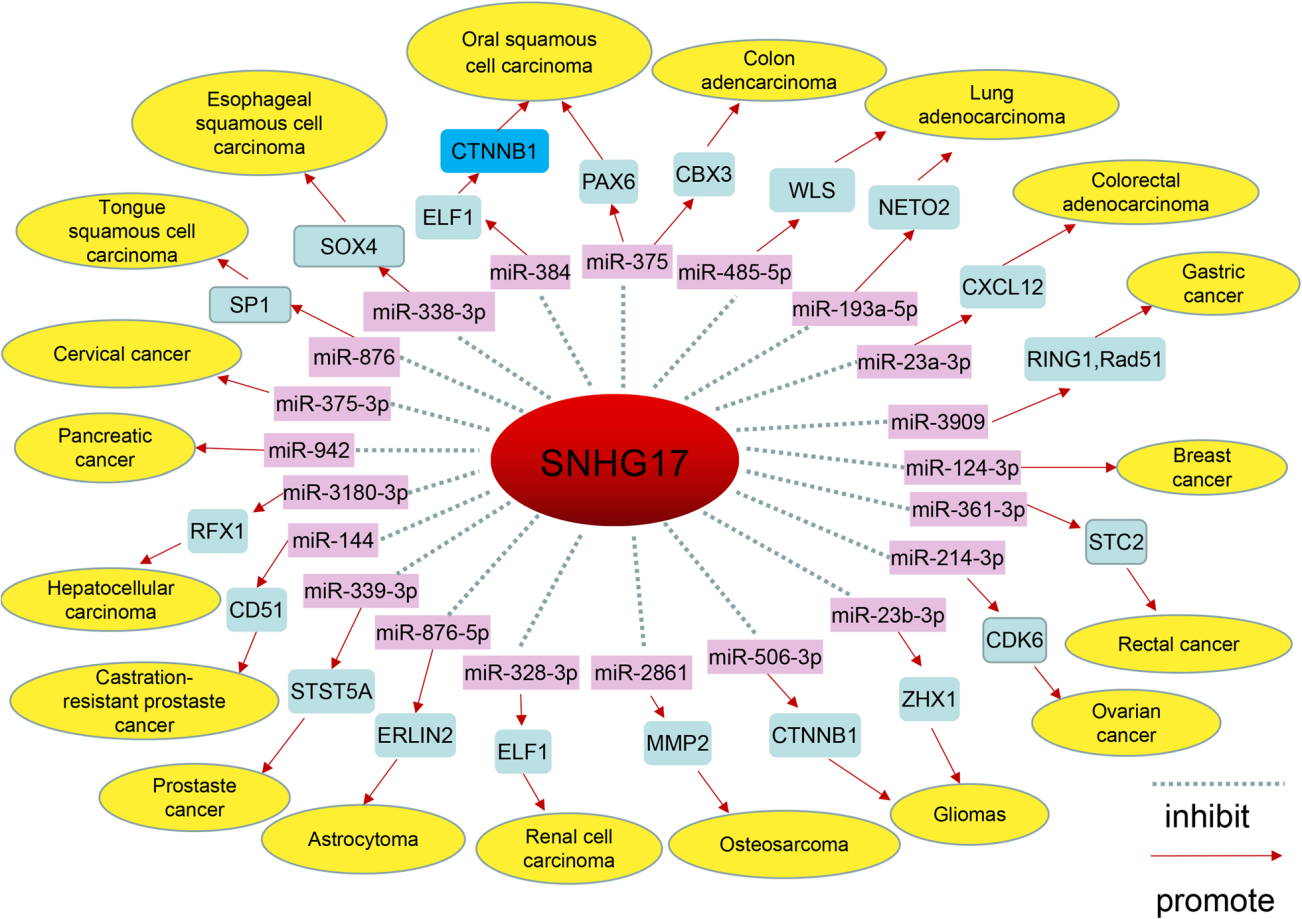
lncRNA SNHG17 function through ceRNA mechanism

While several strands of evidence [63, 79–81] propose that certain ncRNAs may encode micropeptides, the majority of ncRNAs reported to participate in physiological processes do not have the ability to encode proteins [82]. LncRNAs have been widely reported to show abnormal expression in human diseases, in particular in malignant tumors, and they have been suggested to play an important regulatory role in the occurrence, progression, and metastasis of tumors [83]. In this review, we focused on a novel lncRNA from SNHG family, SNHG17, which has been found to be highly expressed in various tumors. SNHG17 has been demonstrated to exhibit a high capacity to modulate the expression of specific targets closely associated with tumor cell proliferation, apoptosis, invasion, migration, amongst others (Table 1). These roles in tumor progression are mediated via a variety of molecular mechanisms frequently related to ceRNA regulation (Fig. 3). For example, SNHG17 exerts a pro-tumor effect in CRC by sponging miR-23a-3p and upregulating CXCL12 [55]. In addition, SNHG17 can combine with LRPRC to decrease the levels of ubiquitylated c-Myc in order to promote cell proliferation [59]. We speculate there may be additional molecular mechanisms related to the action of SNHG17 in tumorigenesis, including methylation [84], acetylation [85], ubiquitination [59, 86], and other epigenetic modifications waiting to be explored.

**Table 1:** Functional characteristics of SNHG17 in various cancers

Cancer Types	Expression	Roles	Assessed Cell Lines	Phenotypes Affected	Related Genes	References
Oral squamous cell carcinoma	UP	Oncogenic	YD-38, SCC-9	Proliferation, cell viability, apoptosis	miR-384, ELF1, CTNNB1, Wnt/β-catenin	[39]
Oral squamous cell carcinoma	UP	Oncogenic	Tca8113, CAL-27	Proliferation, invasion, migration, apoptosis	MiR-375, PAX6	[40]
Tongue squamous cell carcinoma	UP	Oncogenic	Tca8113, CAL-27	Proliferation, invasion, migration, xenograft growth	MiR-876, SP1	[41]
Esophageal squamous cell carcinoma	UP	Oncogenic	TE-1, Eca109	Proliferation, invasion, EMT, xenograft growth	MiR-338-3p, SOX4	[45]
Non-small cell lung cancer	UP	Oncogenic	A549, PC9	Proliferation, migration, apoptosis	FOXA1, XAF1, BIK	[14]
Lung adenocarcinoma	UP	Oncogenic	A549, H1299	Proliferation, migration, invasion, apoptosis	Caspase-3, Bax, Bcl-2, miR-485-5p, WLS	[48]
Lung adenocarcinoma	UP	Oncogenic	A549, H1299	Proliferation, migration, invasion, EMT	MiR-193a-5p, NETO2	[49]
Breast cancer	UP	Oncogenic	MCF-7, MDA-MB-231	Proliferation, migration, invasion	miR-124-3p	[12]
Gastric cancer	UP	Oncogenic	GES-1, SGC-7901, AGS	Repair of DNA damage	NONO, Ku80, miR-3909, Rad51, RING1	[11]
Gastric cancer	UP	Oncogenic	AGS, HGC-27, SGC-7901	Proliferation, invasion, migration, cell cycle, apoptosis, xenograft growth	EZH2, P15, P57	[15]
Colorectal cancer	UP	Oncogenic	DLD-1, HCT116	Proliferation, cell viability, cell cycle, apoptosis, xenograft growth	EZH2, P57	[13]
Rectal cancer	UP	Oncogenic	SW837, SW1463	Proliferation, apoptosis, xenograft growth	MiR-361-3p, STC2	[54]
Colon adenocarcinoma	UP	Oncogenic	HT29, T84	Proliferation, invasion, migration, EMT, xenograft growth	MiR-375, CBX3, E-cadherin, Vimentin, N-cadherin	[53]
Colorectal adenocarcinoma	UP	Oncogenic	RKO, HCT116	Proliferation, migration, cell viability, angiogenesis	MiR-23a-3p, CXCL12	[55]
Hepatocellular carcinoma	UP	Oncogenic	HepG2, Huh7	Proliferation, invasion, migration, EMT, xenograft growth, lung metastasis	E-cadherin, Vimentin, miR-3180-3p, RFX1	[16]
Hepatocellular carcinoma	UP	Oncogenic	HepG2, SF, SNU-449, HCCLM9	Proliferation, cell cycle, xenograft growth, DNA replicating	LRPRC, c-MYc, ppRb, CDK2, CDK4	[59]
Hepatocellular carcinoma	UP	Oncogenic	PLC, SMMC-7721, Hep3B, HuH-7	Proliferation, migration, apoptosis	ERH, TBCA, TDO2, PDK4	[60]
Pancreatic cancer	UP	Oncogenic	PANC-1, AsPC-1	Proliferation, cell viability, invasion, migration, apoptosis, xenograft growth	Caspase-8, Bak, PPARγ, VEGF, ZEB1, BMP, ISG12a, GDNF	[62]
Prostate cancer	UP	Oncogenic	C4-2, LNCaP	Proliferation, cell viability, invasion, apoptosis, xenograft growth, chemotherapeutic resistance	Caspase-3, β-actin, TCF	[66]
Prostate cancer	UP	Oncogenic	PC-3, VCaP	Proliferation, invasion, migration, EMT, apoptosis, xenograft growth	SNORA71B, STAT5A, E-cadherin, N-cadherin	[67]
Castration-resistant prostate cancer	UP	Oncogenic	C4-2, PC-3	Proliferation, invasion, migration, xenograft growth	MiR-144, CD51	[68]
Renal cell carcinoma	UP	Oncogenic	ACHN, 786-O	Proliferation, invasion, migration, cell viability, apoptosis, xenograft growth	MiR-328-3p, H2AX	[64]
Ovarian cancer	UP	Oncogenic	OVCAR-3, PEO1	Proliferation, cell cycle, apoptosis, xenograft growth	STAT3, CTNNB1, miR-214-3p, CDK6	[70]
Ovarian cancer	UP	Oncogenic	OVCAR-3, SKOV3	Proliferation, invasion	FOXA1	[71]
Cervical cancer	UP	Oncogenic	SiHa, HeLa	Proliferation, invasion, migration, apoptosis	MiR-375-3p	[86]
Glioma	UP	Oncogenic	LN229, U251	Proliferation, invasion, migration	MiR-23b-3p, ZHX1	[74]
Glioma	UP	Oncogenic	U87, U251	Proliferation, cell viability, apoptosis, xenograft growth	Caspase3, Caspase9, Bax, YY1, miR-506-3p, CTNNB1, Wnt/β-catenin	[75]
Astrocytoma	UP	Oncogenic	LN215, U138	Proliferation, invasion, migration, stemness, chemotherapeutic resistance	MiR-876-5p, ERLIN2	[76]
Melanoma	UP	Oncogenic	CHL-1, A375	Proliferation, invasion, migration	Caspase3, Caspase9, STAT3, PI3K/AKT	[77]
Osteosarcoma	UP	Oncogenic	CAFs, HOS, SJSA-1	Proliferation, migration,apoptosis, xenograft growth	MiR-2861, MMP2	[18]

## Future perspectives

SNHG17 may possess a substantial value for clinical applications as its expression levels are frequently found to be correlated with tumor size, TNM stages, lymph node metastasis, distant metastasis, and chemical drug resistance (Table 2). In addition, SNHG17 may exhibit potential for early diagnosis in cancers. For instance, SNHG17 can be used to diagnose GC with AUC of 0.748 (P < 0.001) [15], Additionally, the sensitivity and specificity of SNHG17 serum levels for the diagnosis of cervical cancer are 84.7% and 78.2% respectively [73].

**Table 2:** Expression and clinical characteristics of SNHG17 in various cancers

Cancer Types	Sample Size	Expression	Clinical Characteristics	Survival Analysis	References
	Tumor:Normal	Tumor:Normal			
Oral squamous cell carcinoma	40: 40	UP	Advanced TNM stages, lymph node metastasis	No description	[40]
Tongue squamous cell carcinoma	56: 56	UP	Advanced tumor size and TNM stages, lymph node metastasis, diagnosis, poorer OS and prognosis	Kaplan–Meier analysis	[41]
Esophageal squamous cell carcinoma	126: 126	UP	Advanced TNM stages, poorer prognosis	No description	[45]
Lung adenocarcinoma	50: 50	UP	Advanced TNM stages, poorer OS and prognosis	Kaplan–Meier analysis	[49]
Breast cancer	58: 58	UP	Advanced TNM stages, lymph node metastasis, poorer prognosis	Kaplan–Meier analysis	[12]
Gastric cancer	157: 157	UP	Advanced TNM stages, lymph node metastasis, distant metastasis, poorer OS and PFS	Kaplan–Meier analysis, multivariate Cox regression	[51]
Gastric cancer	112: 112	UP	Advanced TNM stages and HP infection, lymph node metastasis, poorer prognosis	Kaplan–Meier analysis	[11]
Gastric cancer	107: 174	UP	Advanced TNM stages, invasion depth, lymph node metastasis, diagnosis	ROC curve	[15]
Colorectal cancer	56: 56	UP	Advanced tumor size and TNM stages, lymph node metastasis, poorer prognosis	Kaplan–Meier analysis	[13]
Rectal cancer	46: 46	UP	No description	No description	[54]
Colon adenocarcinoma	45: 45	UP	No description	No description	[53]
Hepatocellular carcinoma	23: 23	UP	Advanced tumor size and Edmonson-Steiner grades	No description	[16]
Hepatocellular carcinoma	28: 28	UP	Shorter OS and PFS, poorer prognosis	Kaplan–Meier analysis	[59]
Hepatocellular carcinoma	28: 28	UP	Advanced tumor size and TNM stages, shorter OS and DFS, poorer prognosis	Kaplan–Meier analysis, univariate and multivariate Cox regression	[60]
Pancreatic cancer	30: 30	UP	No description	No description	[62]
Prostate cancer	58: 58	UP	Advanced TNM stages, poorer prognosis	Kaplan–Meier analysis	[66]
Prostate cancer	36: 36	UP	Poorer prognosis and PFS	Kaplan–Meier analysis	[67]
Renal cell carcinoma	84: 84	UP	Advanced TNM stages, shorter OS, higher recurrence rate	Kaplan–Meier analysis, univariate and multivariate Cox regression	[64]
Ovarian Cancer	90: 90	UP	Larger tumor size, advanced FIGO stage and histological grade, poorer prognosis	Kaplan–Meier analysis	[70]
Cervical cancer	124: 119	UP	Diagnosis, advanced tumor size and FIGO stage, lymph node metastasis	No description	[86]
Melanoma	148: 148	UP	Lymph node metastasis, advanced tumor stage, poorer prognosis and OS	Kaplan–Meier analysis, multivariate Cox regression	[77]
Osteosarcoma	5: 5	UP	Poorer prognosis and OS	Kaplan–Meier analysis	[18]

In recent years, exosomes [87] have gained increasing interest as mediators of intercellular communication. Exosomes can transport proteins and nucleic acids between cells, several studies [88–90] have demonstrated that tumor-derived exosomes could represent diagnostic, prognostic, or predictive cancer biomarkers, with clinical studies to further explore this currently ongoing. A variety of previous studies have shown that lncRNAs secreted by exosomes can be used for early diagnosis of tumors. For instance, lncRNA-GC1 [91], lncRNA-UEGC1 [92], and lncRNA-HOTTIP [93] found in circulating exosomes could be used to detect early-stage GC, while lncRNA-HOTAIR [94] derived from serum exosomes may serve as novel diagnostic and prognostic biomarker in glioblastoma multiforme. Interestingly, a recent study investigating SNHG17 [18] revealed that exosomal SNHG17 from carcinoma-associated fibroblasts promoted the proliferation and metastasis of osteosarcoma via the miR-2861/MMP2 axis. Therefore, we speculate there may be additional scenarios where tumor-derived exosomes containing SNHG17 are involved in enhancing tumor progression or metastasis.

Therapies targeting ncRNAs, including antisense oligonucleotides (ASOs), small interfering RNAs (siRNAs), short hairpins RNAs (shRNAs), and CRISPR-Cas9-based gene therapy, have been of great interest for clinical management of cancers, and multiple therapies have already been approved by U.S. Food and Drug Administration (FDA) or European Medicines Agency (EMA) [95]. SNHG17 could be a future target for ncRNA-based pan-cancer therapies. In this review, we reported on the aberrant expression of SNHG17 in various cancers. Evidence to date suggests that SNHG17 plays an important role in tumorigenesis and has potential for clinical applications in tumor diagnosis and prognosis, but further studies are needed to unravel all its functions.

## Data Availability

Not applicable.

## Abbreviations

lncRNA: Long non-coding RNA
SNHG17: Small nucleolar RNA host gene 17
WHO: World Health Organization
ceRNA: Competing endogenous RNA
miRNA: MicroRNA
MRE: MicroRNA response element
ncRNA: Non-coding RNA
GEPIA2: Gene Expression Profiling Interactive Analysis 2
OSCC: Oral squamous cell carcinoma
RIP: RNA Binding Protein Immunoprecipitation
ChIP: Chromatin immunoprecipitation
TSCC: Tongue squamous cell carcinoma
qRT-PCR: Quantitative real-time quantitative polymerase chain reaction
TNM: Tumour-node-metastasis
OS: Overall survival
ESCC: Esophageal squamous cell carcinoma
EMT: Epithelial-mesenchymal transition
FISH: Fluorescence in situ hybridization
TCGA: The Cancer Genome Atlas
NSCLC: Non-small cell lung cancer
LUAD: Lung adenocarcinoma
BC: Breast cancer
HP: Helicobacter pylori
PFS: Progression-free survival
DSB: DNA double-strand breaks
CRC: Colorectal cancer
GEO: Gene Expression Omnibus
ENCORI: Encyclopedia of RNA Interactomes
HCC: Hepatocellular carcinoma
snoRNA: Small nucleolar RNA
CRPC: Castration resistant prostate cancer
HSPC: Hormone sensitive prostate cancer
